# Tailoring Hydrogel Sheet Properties through Co-Monomer Selection in AMPS Copolymer Macromers

**DOI:** 10.3390/polym16172522

**Published:** 2024-09-05

**Authors:** Jinjutha Daengmankhong, Thanyaporn Pinthong, Sudarat Promkrainit, Maytinee Yooyod, Sararat Mahasaranon, Winita Punyodom, Sukunya Ross, Jirapas Jongjitwimol, Brian J. Tighe, Matthew J. Derry, Paul D. Topham, Gareth M. Ross

**Affiliations:** 1 Biopolymer Group, Department of Chemistry, Faculty of Science, Naresuan University, Phitsanulok 65000, Thailand; 2 Center of Excellence in Biomaterials, Faculty of Science, Naresuan University, Phitsanulok 65000, Thailand; 3 Department of Chemistry, Faculty of Science, Chiang Mai University, Chiang Mai 50200, Thailand; 4 Center of Excellence in Materials Science and Technology, Chiang Mai University, Chiang Mai 50200, Thailand; 5 Biomedical Sciences Program, Department of Medical Technology, Faculty of Allied Health Sciences, Naresuan University, Phitsanulok 65000, Thailand; 6 Aston Institute for Membrane Excellence, Aston University, Birmingham B4 7ET, UK

**Keywords:** hydrogels, macromers, 2-Acrylamido-2-methyl-1-propanesulfonic acid sodium salt, photosensitizer, biomedical applications

## Abstract

This study investigates hydrogels based on 2-Acrylamido-2-methyl-1-propanesulfonic acid sodium salt (AMPS) copolymers, incorporating N-hydroxyethyl acrylamide (HEA) and 3-sulfopropyl acrylate potassium salt (SPA). The addition of HEA and SPA is designed to fine-tune the hydrogels’ water absorption and mechanical properties, ultimately enhancing their characteristics and expanding their potential for biomedical applications. A copolymer of AMPS, 2-carboxyethyl acrylate (CEA) combined with methacrylic acid (MAA) as poly(AMPS-stat-CEA-stat-MAA, PACM), was preliminarily synthesized. CEA and MAA were modified with allyl glycidyl ether (AGE) through ring-opening, yielding macromers with pendant allyl groups (PACM-AGE). Copolymers poly(AMPS-stat-HEA-stat-CEA-stat-MAA) (PAHCM) and poly(AMPS-stat-SPA-stat-CEA-stat-MAA) (PASCM) were also synthesized and modified with AGE to produce PAHCM-AGE and PASCM-AGE macromers. These copolymers and macromers were characterized by ^1^H NMR, FT-IR, and GPC, confirming successful synthesis and functionalization. The macromers were then photocrosslinked into hydrogels and evaluated for swelling, water content, and mechanical properties. The results revealed that the PASCM-AGE hydrogels exhibited superior swelling ratios and water retention, achieving equilibrium water content (~92%) within 30 min. While the mechanical properties of HEA and SPA containing hydrogels show significant differences compared to PACM-AGE hydrogel (tensile strength 2.5 MPa, elongation 47%), HEA containing PAHCM-AGE has a higher tensile strength (5.8 MPa) but lower elongation (19%). In contrast, SPA in the PASCM-AGE hydrogels led to both higher tensile strength (3.7 MPa) and greater elongation (92%), allowing for a broader range of hydrogel properties. An initial study on drug delivery behavior was conducted using PACM-AGE hydrogels loaded with photosensitizers, showing effective absorption, release, and antibacterial activity under light exposure. These AMPS-based macromers with HEA and SPA modifications demonstrate enhanced properties, making them promising for wound management and drug delivery applications.

## 1. Introduction

Hydrogels, defined as three-dimensional (3D) networks formed through the physical or chemical crosslinking of hydrophilic polymers, are a versatile class of materials extensively used in the biomedical field. Their applications include soft tissue engineering (such as skin, muscles, and blood vessels), drug delivery systems, and wound dressings [1]. This versatility arises from their ability to retain large amounts of water due to their hydrophilic nature, and their tunable chemical, mechanical, and biological properties, which allow them to mimic the extracellular matrix (ECM) of biological tissues [2,3,4,5]. Among these applications, hydrogel wound dressings stand out due to their ability to maintain a moist environment, protect against infections, and promote healing [6,7]. However, designing the ideal hydrogel dressing to meet specific requirements remains a challenge in modern medical technology, especially mechanical properties of high-water-content materials.

The polymers used for hydrogel dressings can be classified mainly into two categories: natural polymers (such as chitosan, silk fibroin, collagen, hyaluronic acid, and gelatin) and synthetic polymers (such as polyurethane, poly(vinyl alcohol) (PVA), poly(lactide-*co*-glycolide) (PLGA), poly(acrylic acid) (PA), poly(2-hydroxyethyl methacrylate) (PHEMA), poly(acrylamide), and poly(2-acrylamido-2-methyl propane sulfonic acid) (PAMPS)) [5,8]. In general, natural polymers provide superior biocompatibility, whereas synthetic polymers offer enhanced mechanical strength and customizable properties [9,10]. Among synthetic polymers, PAMPS shows great promise due to its active functional groups. The presence of a sulfonate group in AMPS residues resembles the glycosaminoglycan present in the skin’s extracellular matrix, which plays a key role in maintaining and providing moisture to the body [11]. Other monomers used for copolymerization with AMPS include acrylic acid, acrylamide, 2-hydroxyethyl acrylate [12,13,14], methacrylic acid, *N*,*N*-dimethylacrylamide [15] methyl methacrylate, and 2-hydroxyethyl methacrylate [16]. However, reported AMPS hydrogels often exhibit poor mechanical properties due to their low cohesive force [17,18]. In our previous work [19], we synthesized copolymers of AMPS using acidic monomers (2-carboxyethyl acrylate (CEA) and methacrylic acid (MAA)) to form poly(AMPS-*stat*-CEA-*stat*-MAA) (PACM) copolymers. This PACM copolymer was further modified using a ring-opening reaction with allyl glycidyl ether (AGE) to generate poly(AMPS-*stat*-CEA-*stat*-MAA)-*graft*-AGE (PACM-AGE) macromer, which showed rapid hydrogel synthesis using UV-initiated crosslinking with poly(ethylene glycol) diacrylate (PEGDA). This process produced hydrogels within 10 s, significantly faster than regular vinyl monomers [19].

Herein, we have significantly advanced our previous work on AMPS-based macromers by exploring the effects of copolymerizing AMPS with 3-sulfopropyl acrylate potassium salt (SPA) or *N*-hydroxyethyl acrylamide (HEA). The primary motivation was to use these co-monomers to tune the water absorption and mechanical properties of the hydrogels, ensuring they do not become overly absorbent and prone to breaking. SPA was chosen for its ionic nature and ability to stabilize acrylic dispersions, which enhance the mechanical strength and water retention of the hydrogels [20,21,22,23]. HEA was selected as a non-ionic hydrophilic monomer, reducing swellability whilst providing long-term antifouling and durability suitable for biomedical applications [24,25]. By incorporating SPA or HEA, we aimed to balance the mechanical integrity of the hydrogels with their water absorption capability, making them more robust and suitable for practical applications.

**Figure 1:**
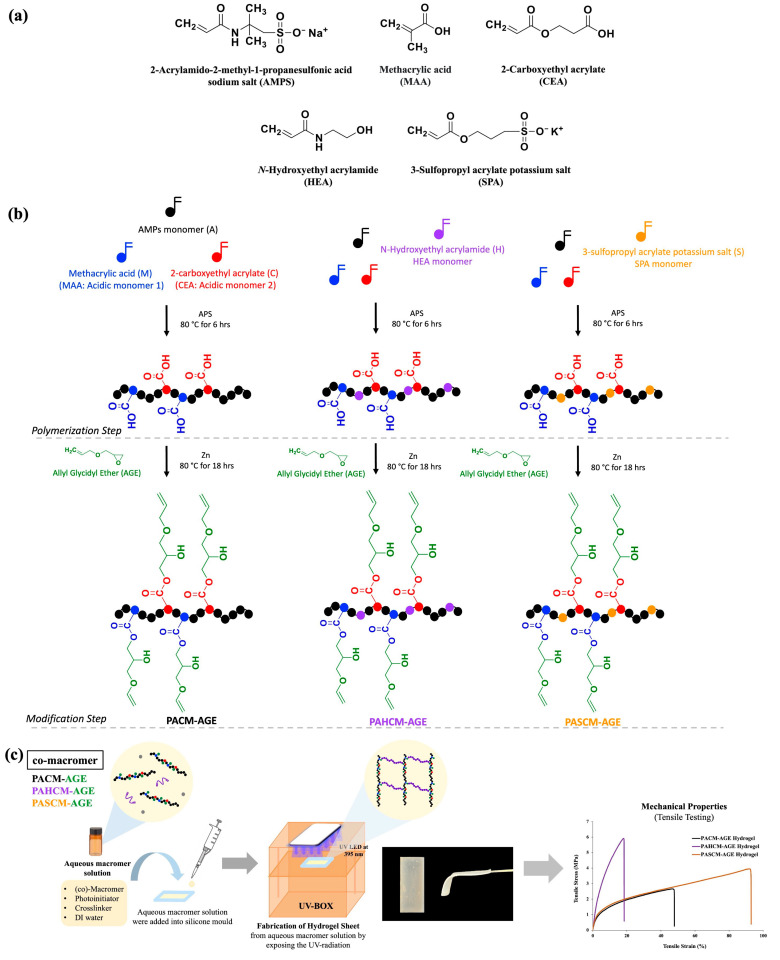
(**a**) Chemical structures of monomers, (**b**) schematic diagram of the synthesis of statistical copolymers (PACM, PAHCM, and PASCM) and macromers (PACM-AGE, PAHCM-AGE, and PASCM-AGE) via free-radical polymerization, and (**c**) the fabrication of hydrogel sheets from different co-macromers by photopolymerization along with their tensile properties.

The successful synthesis of copolymers and macromers was confirmed using a suite of analytical techniques before being used to fabricate hydrogel dressing sheets using free-radical polymerization with UV-initiated crosslinking using PEGDA (see Figure 1). The hydrogel sheets, derived from AMPS-based macromers, were assessed for their mechanical properties, water content, swelling ratios, and morphologies. Additionally, we incorporated water-soluble dyes (Rose Bengal and Methylene Blue) to study the uptake and release profiles of these photosensitizing agents and assessed antibacterial properties against Gram-positive and Gram-negative bacteria. Our findings suggest that the innovative design of these AMPS-based macromer hydrogels presents a promising avenue for drug delivery systems, with particular benefits for enhancing wound-management techniques.

## 2. Materials and Methods

### 2.1. Materials

2-Acrylamido-2-methyl-1-propanesulfonic acid sodium salt (AMPS) 50 wt.% in H_2_O, 2-carboxyethyl acrylate (CEA), methacrylic acid (MAA), *N*-hydroxyethyl acrylamide (HEA), 3-sulfopropyl acrylate potassium salt (SPA), ammonium persulfate (APS), allyl glycidyl ether (AGE), zinc, poly(ethylene glycol) diacrylate (PEGDA) (*M*_n_ = 575 g/mol), diphenyl(2,4,6-trimethylbenzoyl phosphine oxide) (TPO), Rose Bengal (RB), phosphate buffer saline (PBS) tablets, and acetone were purchased from Sigma-Aldrich (Gillingham, UK). The Methylene Blue (MB), AR grade, used was from Q RëC™ (Bangkok, Thailand).

### 2.2. Synthesis of AMPS-Based Copolymers and AMPS-Based Macromers

Three different co-polymers based on AMPS were first synthesized (see Table 1), following our previous work [19]. Briefly, a copolymer of AMPS, CEA, and MAA was synthesized as a control sample via free-radical polymerization, using APS as a thermal initiator in water at a temperature of 80 °C for a duration of 6 h, while maintaining a stirring speed of 320 rpm. This procedure allowed for the formation of a *stat*istical copolymer of poly(AMPS-*stat*-CEA-*stat*-MAA) (PACM) [19]. Two additional copolymers were synthesized by the addition of either HEA or SPA into the polymerization system utilizing the same method of synthesis as PACM. The additional copolymers were defined as poly(AMPS-*stat*-HEA-*stat*-CEA-*stat*-MAA) (PAHCM) and poly(AMPS-*stat*-SPA-*stat*-CEA-*stat*-MAA) (PASCM), respectively.

### 2.3. Synthesis of AMPS-Based Macromers

The AMPS-based macromers of PACM-AGE, PAHCM-AGE, and PASCM-AGE were synthesized through the transformation of the pendant acid groups of CEA and MAA into pendant allyl groups, following our previous work [19]. Briefly, the PACM, PAHCM, and PASCM copolymers were reacted with AGE, using zinc as a catalyst at 80 °C for 18 h with a continuing stirred speed of 320 rpm in water (see Table 1). After the completion of the reaction, the resultant macromer solution was added dropwise into acetone to precipitate and to purify the synthesized macromers before the elimination of residue acetone, first by decanting and then by rotary evaporator, and finally dried in a hot air oven at 60 °C for 24 h before use.

### 2.4. Fabrication of Hydrogel Sheet

Hydrogel sheets for all macromers, PACM-AGE, PAHCM-AGE, and PASCM-AGE, were fabricated through a conventional photo-initiated free-radical crosslinking system. A total of 0.2 g of macromer was mixed with 0.1 g of PEGDA (*M*_n_ = 575 g mol^−1^) (as the crosslinker) and 0.002 g of TPO (as the photo-initiator) with 0.6 mL of deionized water as the solvent (water content = 66.67%). This mixture was shaken using an orbital shaker at a speed of 30 rpm for 24 h to promote a homogeneous mixture solution before exposing with UV radiation at a wavelength of 395 nm with an intensity of 425 mW cm^−2^ in a silicone mold (5 cm × 3 cm).

### 2.5. Characterization of Copolymers and Macromers

#### 2.5.1. Chemical Structures: ^1^H NMR Spectroscopy

The chemical structures of copolymers and macromers were analyzed by proton (^1^H) nuclear magnetic resonance (NMR) spectroscopy using a Bruker 300 MHz UltraShield cryomagnet with an Advance Neo console (Bruker, Coventry, UK), with 128 scans and 256 scans on average per spectrum. The non-crosslinked samples were prepared by dissolving them in D_2_O. To ensure complete dissolution, the samples were thoroughly mixed before analysis, which was conducted at room temperature (25 °C). A concentration range of 0.05–0.10 mg/mL was used for the examination.

#### 2.5.2. Chemical Functional Groups: FT-IR Spectroscopy

The functional groups of copolymers and macromers were tested by Fourier transform infrared (FT-IR) spectroscopy using a PerkinElmer Spectrum Two spectrometer (Waltham, MA, USA) with UATR at 4000–400 cm^−1^. All samples were kept dried at room temperature before testing.

#### 2.5.3. Molecular Weight: GPC

Molecular weight distributions of copolymers and macromers were assessed by gel permeation chromatography (GPC) using water (0.05% *w*/*v* NaN_3_) as the eluent, and the flow rate was fixed at 1.0 mL min^−1^. Two columns of Agilent (Agilent 1260 Infinity II system (Agilent, Stockport, UK) PL Aquagel-OH mixed-H, 7.5 × 300 mm, 8 μm, with a PL aquagel guard column (molecular weight in ranges of 6000–10,000,000 Da) in place were set up with temperature of 35 °C and an RI detector. A PEG/PEO nominal *M*_p_ 106–1,500,000 Da (PL2080-0201, Agilent, Stockport, UK) was performed for calibration.

### 2.6. Characterization of Hydrogel Sheets

#### 2.6.1. Equilibrium Water Content

The percentage of equilibrium water content (%EWC) was conducted using a gravimetric technique. The swollen hydrogel sheets (W_s_) were weighed and then dehydrated in an oven at 60 °C for 72 h. Then, the dehydrated hydrogel sheets (W_d_) were weighed. The %EWC was reported until the weight of the samples reached to a constant value. Each hydrogel sheet was measured three times under the same conditions and the average values were reported using the following equation:

%EWC = ((W_s_ − W_d_)/W_s_) × 100%

where W_s_ and W_d_ are the swollen and dried weights of the hydrogel sheets, respectively.

#### 2.6.2. Differential Scanning Calorimetry (DSC)

The water structure in the hydrogel samples was analyzed using differential scanning calorimetry (DSC) (Mettler Model DSC1, Greifensee, Switzerland) to determine the percentage of freezing water and non-freezing water. The DSC heating curves of all hydrogel samples exhibited endothermic peaks between −10 and 20 °C, corresponding to the melting of various forms of water that had frozen during the cooling phase [26]. For sample preparation, swollen hydrogels at their equilibrium water content were cut into pieces using a cork borer and weighed (~12 mg), then sealed in aluminum pans to prevent water evaporation. This procedure was repeated for each sample in triplicate (N = 3). The pans were placed in the sample holder of the thermal analyzer, and the samples were scanned using the following parameters:

Cool from 25.0 °C to −70.0 °C, hold for 5 min at −70.0 °C, heat from −70.0 °C to −25.0 °C at 20.00 °C/min, and heat from −25.0 °C to 25.0 °C at 10.00 °C/min.

The area under the endothermic peak(s) represents the energy needed to melt the freezing water within the sample. Given the known sample weight and the specific energy required to melt 1 g of pure water (333.5 Jg^−1^) [27], the calculation of the percentage of freezable (free) water in the sample was used based on Equation (1).

Free water (%) = [∆HTr/(m × ∆Hf)] × 100(1)

where ∆Hf = 333.5 J/g, ∆HTr = heat of transition, and m = sample weight (mg). The amount of non-freezing water was obtained by subtracting the amount of freezing water from the total percent water content following Equation (2).

Non-freezing water content (%) = Total water content (%) − Free water content (%).(2)

#### 2.6.3. Swelling Test

The swelling behavior of the hydrogel sheets was studied by completely immersing the samples in de-ionized water at room temperature. The swollen hydrogel sheets were taken out from the de-ionized water, and excess water at the surface was removed and weighed at each time interval, ranging from 1 to 180 min. The % swelling was calculated based on the change in weight using the following equation:

$$\%swelling=\frac{W_{f}-W_{i}}{W_{i}}\times 100\%$$

where W_i_ and W_f_ are the initial weight and final weight at different times, respectively. The samples were measured three times for each sample and reported as the average % swelling with the standard deviation.

#### 2.6.4. Tensile Testing

The tensile stress and percentage of elongation at break of the hydrogel sheet samples (at a fabricated hydrogel water content of 66.67%) were tested following ASTM D638 [28], type 5. The specimen dimensions were a gauge length of 7.62 mm, a specimen width in the gauge length area of 3.18 ± 0.5 mm, and a thickness of 2.5 mm. The hydrogel sheets were cut into a dog-bone shape and stored at room temperature before testing. Testing was conducted at room temperature using an Instron 5965 (Norwood, MA, USA) with pneumatic side-action grips (Instron 2712-04x series, 1 kN model, Norwood, MA, USA) and at a crosshead speed set to 30 mm/min. Each hydrogel sample was tested six times to determine the mean and standard deviation values.

#### 2.6.5. Dye Uptake and Release

The hydrogel sheet of PACM-AGE was chosen to study the release profiles using two different water-soluble photosensitizer dyes: Rose Bengal (RB) and Methylene Blue (MB). The PACM-AGE hydrogel sheet was soaked in 1.0 mL of dye solution with a concentration of 0.001 M, following the minimum inhibitory concentration (MIC) guidelines, for 30 min to reach equilibrium before testing dye release [29] and MB [30]. After that, the hydrogels containing the absorbed dye were placed into vials containing 1 mL of Phosphate Buffer Saline (PBS). The release times for each dye were studied at intervals of 1, 2, 3, 4, 5, 6, 12, 16, 18, and 24 h. After each time period, the solution was removed, and 1 mL of fresh PBS was added to the vial for the next interval. The amount of dye released was measured in triplicate (n = 3) using UV absorbance with a multiplate reader (Biotek, Model Synergy H1 Hybrid Reader, Santa Clara, CA, USA). The wavelengths used were 550 nm for Rose Bengal and 668 nm for Methylene Blue. The absorbance of the solution at each time point was converted to concentration using calibration curves for both dyes. To assess dye absorption in the hydrogels, UV absorbance of the dye solution was measured both before and after the uptake process using calibration curves.

#### 2.6.6. In Vitro Anti-Bacterial Experiments

The antimicrobial activities of the PACM-AGE hydrogel sheet incorporated with Rose Bengal (RB) and Methylene Blue (MB) were examined using the disk diffusion method according to Clinical and Laboratory Standards Institute (CLSI) guideline M02-A11 [31]. *Staphylococcus aureus* (*S. aureus*) ATCC 25923 and *Escherichia coli* (*E.coli*) ATCC 25922 were obtained from the American Type Culture Collection (ATCC; Manassas, VA, USA). The turbidity of the inoculum was adjusted to 0.5 McFarland standard around 1–2 × 10^8^ CFU/mL [32]. For the quality-control (QC) group, an antibiotic disk 10 microgram Gentamicin (CN) was used as a control for *Staphylococcus aureus* (*S. aureus*) ATCC 25923 and *Escherichia coli* (*E. coli*) ATCC 25922. The hydrogel samples were cut into 6 mm diameter disks using a cork borer to ensure consistent size. The bacterial isolates were steaked on Mueller–Hinton Agar (MHA, Oxoid, Basingstoke, UK) plates with the samples, and were incubated at approximately 37 °C for 18 h. The inhibition zones were measured in diameter (mm) using a vernier caliper.

## 3. Results and Discussion

The effects of incorporating *N*-hydroxyethyl acrylamide (HEA) and 3-sulfopropyl acrylate potassium salt (SPA) into AMPS-based hydrogel sheets were studied. PACM-AGE hydrogels inherently absorb a substantial amount of water due to osmotic pressure, which can result in inferior mechanical properties. This research aims to enhance the performance characteristics of hydrogels for potential biomedical applications. By introducing HEA and SPA, the goal is to improve the hydrogels’ mechanical strength and water retention, which are critical for applications such as wound dressing and drug delivery systems. The incorporation of non-ionic monomers such as HEA mitigates this issue by reducing the extent of swelling, thereby preserving the structural integrity of the hydrogel at equilibrium water content (EWC). Conversely, the addition of another type of anionic monomer, such as SPA, alters the mechanical properties differently. Initially, the copolymers poly(AMPS-*stat*-CEA-*stat*-MAA) (PACM), poly(AMPS-*stat*-HEA-*stat*-CEA-*stat*-MAA) (PAHCM), and poly(AMPS-*stat*-SPA-*stat*-CEA-*stat*-MAA) (PASCM) were synthesized. These copolymers were then modified by reacting their acid functional groups with AGE, producing three distinct macromers: poly(AMPS-*stat*-CEA-*stat*-MAA)-graft-AGE (PACM-AGE), poly(AMPS-*stat*-HEA-*stat*-CEA-*stat*-MAA)-graft-AGE (PAHCM-AGE), and poly(AMPS-*stat*-SPA-*stat*-CEA-*stat*-MAA)-graft-AGE (PASCM-AGE). All copolymers and macromers were characterized to confirm their chemical structure, functional groups, and molecular weight. These macromers were subsequently formed into hydrogels using conventional free-radical crosslinking. The resulting hydrogels were then tested for their swelling behavior, water structure and content, mechanical properties (tensile stress and elongation), photosensitizing agent release profiles, and antibacterial activity.

### 3.1. Copolymers and Macromers

#### 3.1.1. Chemical Structure Verification by ^1^H NMR Spectroscopy

**Figure 2:**
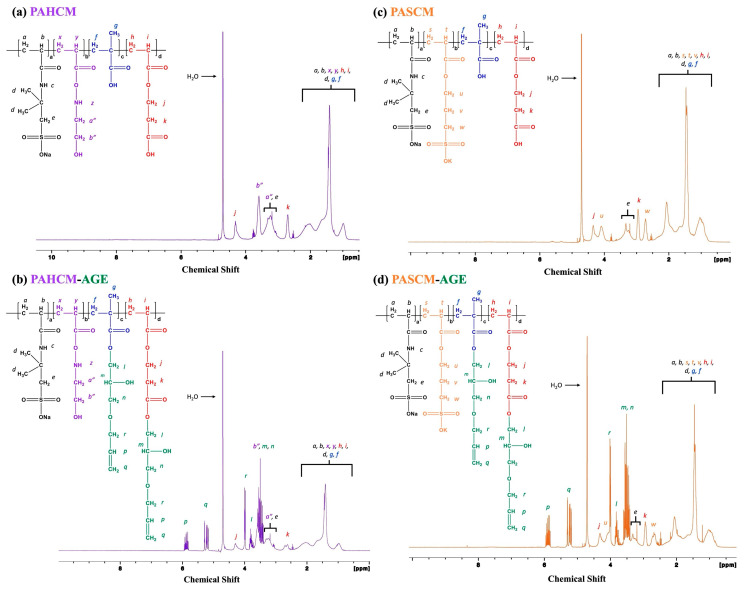
^1^H NMR spectra of (**a**) PAHCM copolymers, (**b**) PAHCM-AGE macromer, (**c**) PASCM copolymer, and (**d**) PASCM-AGE macromer.

The ^1^H NMR spectra of the copolymers (PAHCM and PASCM) and macromers (PAHCM-AGE and PASCM-AGE) (Figure 2) indicate changes in the proton environments before and after polymerization and functionalization. The copolymer structures of PAHCM and PASCM are confirmed in Figure 2a,c. The epoxide groups of AGE reacted with the pendant carboxylic acid groups of acidic monomers, resulting in polymer chains with pendant allyl groups, as confirmed by ^1^H NMR spectroscopy in Figure 2b (PAHCM-AGE macromer) and Figure 2d (PASCM-AGE macromer). The signals of the attached allyl groups from the modification with AGE were observed at 4.0 (*r*), 5.2 (*q*), and 5.8 (*p*) ppm. For the PACM copolymer and PACM-AGE macromer, the ^1^H NMR spectra showed the same chemical shifts as observed in our previous work [19].

#### 3.1.2. Chemical Structure Verification by FT-IR Spectroscopy

**Figure 3:**
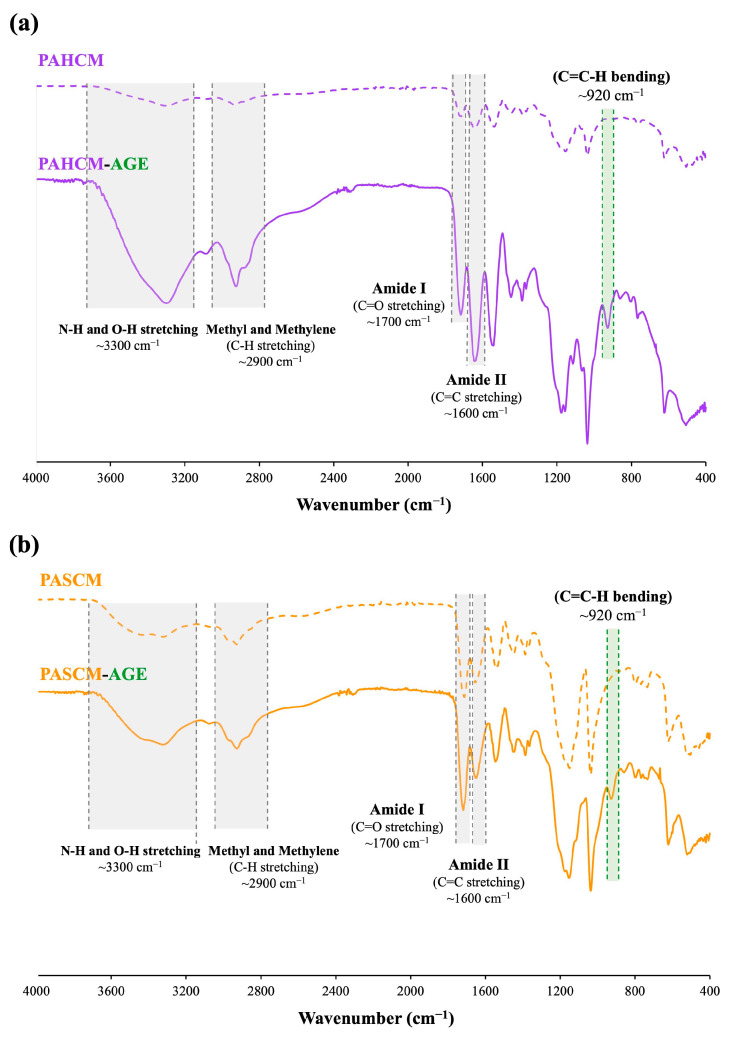
FT-IR spectra of (**a**) PAHCM copolymer and PAHCM-AGE macromer and (**b**) PASCM copolymer and PASCM-AGE macromer.

FT-IR spectroscopy was additionally employed to confirm the successful modification of AGE onto the copolymers of PAHCM and PASCM (Figure 3). The FT-IR spectra of both copolymers and macromers exhibited absorption bands at 3300 and 2900 cm^−1^, corresponding to N–H and O–H stretching, as well as methyl and methylene groups (C–H stretching). Additionally, bands around 1600 and 1700 cm^−1^ were observed, attributed to amide I (C=O stretching) and amide II (C=C stretching), respectively. The FT-IR spectra revealed differences in the characteristic bands of the copolymers (PAHCM and PASCM) before functionalization and the macromers (PAHCM-AGE and PASCM-AGE) after functionalization with AGE. The macromers exhibited new bands corresponding to allyl groups, notably the appearance of C=C–H bending around 920 cm^−1^ and O–H bending (out-of-plane) at 900–860 cm^−1^, derived from the opened epoxide ring of AGE [33]. This observation confirmed the successful attachment of allyl groups from AGE within the co-macromer structure. The FT-IR spectra of the PACM copolymer and PACM-AGE macromer also demonstrated the presence of C=C–H bending belonging to allyl groups, consistent with our previous work [19].

#### 3.1.3. Molar Mass Analysis by GPC

**Figure 4:**
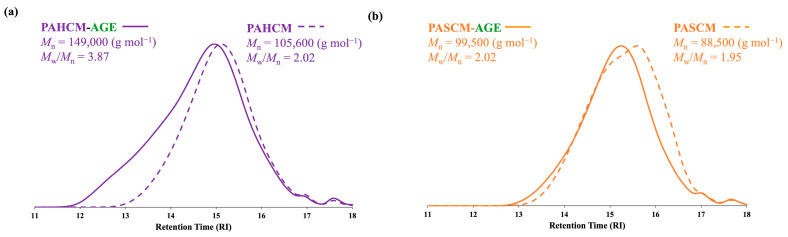
GPC traces of (**a**) PAHCM copolymer and PAHCM-AGE macromer, and (**b**) PASCM copolymer and PASCM-AGE macromer.

GPC analysis was conducted to further validate the effective synthesis of the macromers. The macromers (both PAHCM-AGE and PASCM-AGE) demonstrated an increase in molecular weight compared to their respective copolymers (PAHCM and PASCM) (Figure 4). The shift of the peak towards a lower retention time indicated an increase in molar mass after functionalization with AGE, which was consistent with expectations. Specifically, for PAHCM, the molar mass increased from 105,600 g mol^−1^ (dispersity, *Đ* = *M*_w_/*M*_n_ ~ 2.02) to 149,000 g mol^−1^ (*Đ* ~ 3.87) after modification to PAHCM-AGE. For PASCM, the molar mass increased from 88,500 g mol^−1^ (dispersity, *Đ* = *M*_w_/*M*_n_ ~ 1.95) to 99,500 g mol^−1^ (*Đ* ~ 2.02) after the modification to PASCM-AGE, whereas the molecular weight of PACM showed at 103,400 g mol^−1^ (dispersity, *Đ* = *M*_w_/*M*_n_ ~ 1.72) and PACM-AGE showed at 136,500 g mol^−1^ (dispersity, *Đ* = *M*_w_/*M*_n_ ~ 3.28) [19]. The analysis of NMR, FT-IR, and GPC all show the distinctive features of the successful addition of HEA and SPA into the system and copolymerization to form PAHCM-AGE and PASCM-AGE. From the shoulder peak and increase in both *M*_n_ and *M*_w_/*M*_n_ in PAHCM-AGE compared to PASCM-AGE, it is proposed that the PAHCM-AGE contains crosslinked partial macromers (as seen with PACM-AGE in our previous work), whereas PASCM-AGE, only presents an increase in *M*_n_ in line with the addition of AGE.

### 3.2. Hydrogel Sheets

The next step to assess the newly synthesized PAHCM-AGE and PASCM-AGE macromers was to fabricate them into hydrogel sheets via conventional free-radical polymerization (crosslinking) in the presence of PEGDA (*M*_n_ = 575 g mol^−1^) as the crosslinker. These hydrogel sheets were evaluated for their mechanical properties, morphology, and swelling behavior. Hydrogel sheets fabricated from PACM-AGE, with and without PEGDA, were used as control samples to assess the effects of HEA and SPA in the macromer compositions. Additionally, drug-model release profiles and antibacterial performance of two photodynamic agents were assessed on the control hydrogel (PACM-AGE), as these hydrogels are potential candidates for the transdermal delivery of active agents.

#### 3.2.1. Hydrogel Swelling Behavior and Morphology

**Figure 5:**
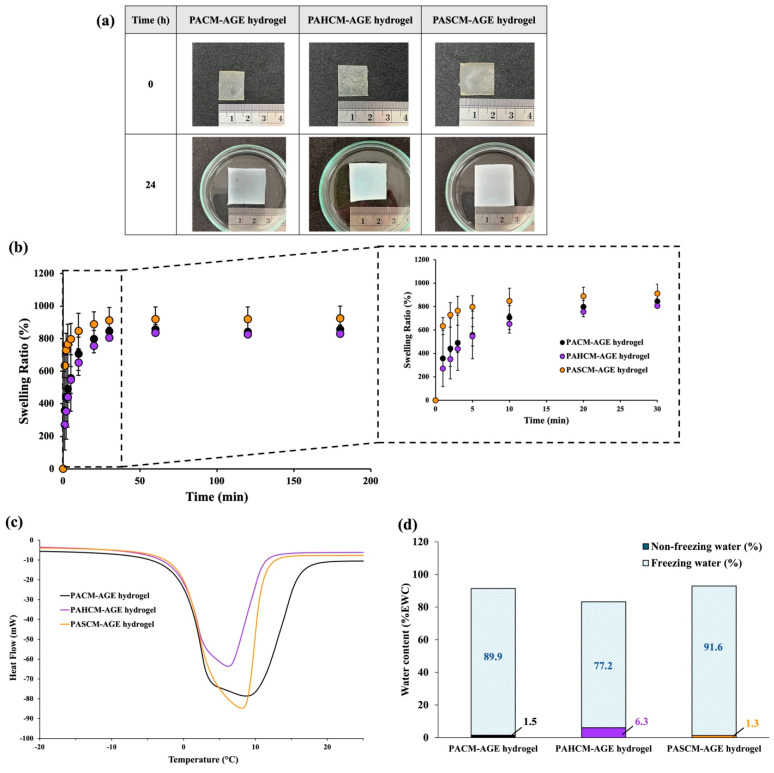
Optical images (**a**), swelling ratio (**b**), DSC thermograms, (**c**) and % total water content, showing mass ratios of non-freezing and freezing water (**d**) of crosslinked hydrogel sheets of PACM-AGE, PAHCM-AGE, and PASCM-AGE.

Since the water in hydrogels often considerably influences their final properties, we initially assessed the water content, water behavior, and swelling ratio of the crosslinked hydrogel sheets of PACM-AGE, PAHCM-AGE, and PASCM-AGE (Figure 5). The crosslinked PASCM-AGE hydrogel exhibited the highest swelling within 30 min, with an increase of approximately 1.7 times in length and width, and 1.6 times in thickness compared to the non-soaked sample (Figure 5a). The swelling ratios of all hydrogels were observed and compared (Figure 5b). It was found that all hydrogels absorbed water rapidly within the first 5 min, followed by a slower absorption rate until reaching equilibrium water content within 30 min. Notably, the crosslinked PASCM-AGE hydrogel absorbed greater quantities of water than the PACM-AGE and PAHCM-AGE hydrogels, respectively. The equilibrium water content measurements showed that the crosslinked PASCM-AGE hydrogel exhibited a water content of ~92%, which is higher than that of PACM-AGE (~89%) and PAHCM-AGE (~77%) (Figure 5d). The mass ratios of freezing to non-freezing water, as assessed from the DSC isotherms (Figure 5c), are 71:1 for PASCM-AGE hydrogel, 12:1 for PAHCM-AGE hydrogel, and 61:1 for PACM-AGE hydrogel. These ratios demonstrate that the PASCM-AGE hydrogel had more water molecules present between the polymer chains (free water), enabling water retention within the hydrogel networks. Conversely, the lower mass ratio in the PAHCM-AGE hydrogel suggests a tighter network structure with extensive hydrogen bonding within the hydrogels. These results align with expectations, as SPA, containing sulfopropyl potassium salt, has a greater affinity for attracting water compared to PACM-AGE and PAHCM-AGE. In contrast, HEA does not contain any sulfonate groups, resulting in the lowest water content and swelling performance. This supports our initial motivation to tune the water content and mechanical properties of the hydrogels by incorporating specific co-monomers.

**Figure 6:**
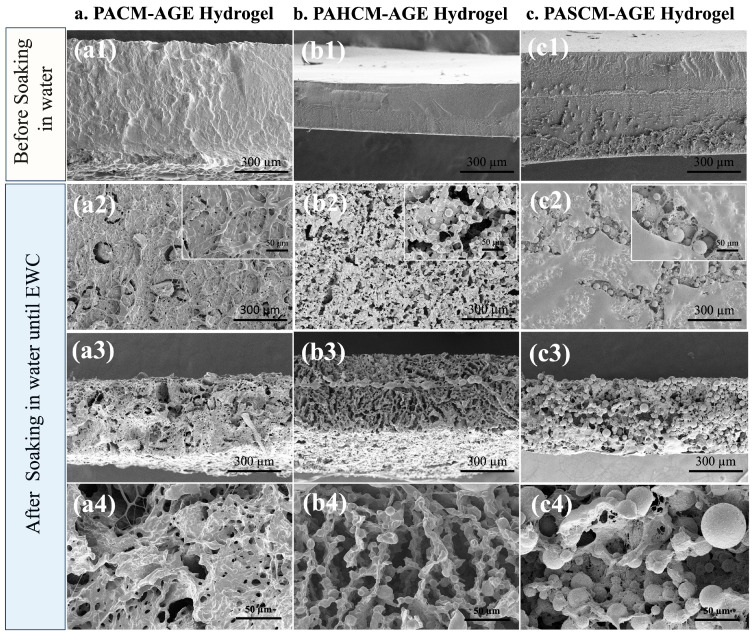
FE-SEM images of crosslinked hydrogel sheets of (**a**) PACM-AGE, (**b**) PAHCM-AGE, and (**c**) PASCM-AGE before soaking in water ((**a1**,**b1**,**c1**)—cross-sectioned images at magnification of 200×) and after soaking in water until equilibrium water content (EWC) ((**a2**,**b2**,**c2**)—surface image at magnification of 200× and 500× (inset images); (**a3**,**b3**,**c3**)—cross-sectioned images at magnification of 200×; (**a4**,**b4**,**c4**)—cross-sectioned images at magnification of 500×).

Additionally, the morphology of all crosslinked hydrogel sheets was examined both after polymerization (before soaking in water) and after soaking in water until they reached equilibrium water content. The samples were then freeze-dried and analyzed using FE-SEM, with the results shown in Figure 6. A dense surface morphology was observed in all hydrogels before soaking in water (Figure 6(a1,b1,c1)). After soaking the hydrogels to their EWC, an increase in porosity was observed in the dense structure of the PACM-AGE hydrogel due to the removal of water molecules contained within the gels (Figure 6(a2–a4)). In contrast, a series of interconnecting droplets (or fused-sphere morphology) with macroporous structures (voids between the spheres forming a continuous porous space) were observed in the PAHCM-AGE (Figure 6(b2–b4)) and PASCM-AGE (Figure 6(c2–c4)) hydrogels. In this case, the excess water molecules in the hydrogel networks could trigger the formation of fused-sphere morphology and pores, leading to phase separation. Additionally, the PAHCM-AGE hydrogel exhibited a more regular phase separation with smaller fused-sphere morphology, larger voids, and less swelling in water compared to PASCM-AGE. This difference is attributed to the effects of HEA and SPA molecules, as HEA, with its molecular structure, has a greater capacity for hydrogen bonding, inducing different behaviors in the crosslinked polymer networks within the hydrogels. This result was also observed in the study of water content and in the ratio of freezing to non-freezing water from the DSC thermograms (Figure 5c,d).

#### 3.2.2. Mechanical Performance of Hydrogels

**Figure 7:**
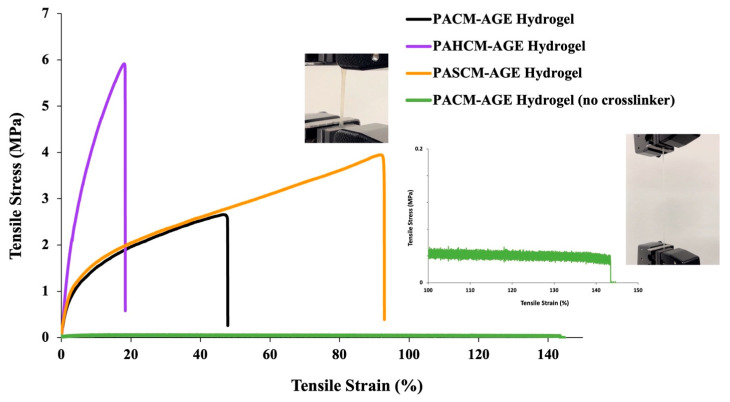
Tensile strength and percentage of elongation of crosslinked hydrogel sheets of PAHCM-AGE, PASCM-AGE, and PACM-AGE fabricated using PEGDA (*M*_n_ = 575, crosslinker), and PACM-AGE fabricated without crosslinker.

One reason for incorporating HEA and SPA into the macromer was to enhance the mechanical performance of the resulting hydrogel sheets, in line with our overall goal of tuning the water content and mechanical properties to ensure durability and prevent excessive water absorption. Figure 7 shows the tensile strength and percentage of tensile elongation of all hydrogel sheets. The crosslinked PACM-AGE hydrogel sheet (hydrogel as fabricated as control sample) exhibits a tensile elongation of approximately 47% with a tensile strength of 2.5 MPa. When HEA (H) and SPA (S) were incorporated into the macromer chains, as in PAHCM-AGE and PASCM-AGE, respectively, considerable changes in tensile strength and elongation were observed. Specifically, the inclusion of HEA in the PAHCM-AGE hydrogel sheet resulted in a higher tensile strength (approximately 5.8 MPa) but lower elongation (approximately 19%) compared to the crosslinked PACM-AGE hydrogel. In contrast, the inclusion of SPA in the PASCM-AGE hydrogel enhanced both tensile strength (approximately 3.7 MPa) and elongation (approximately 92%) compared to the crosslinked PACM-AGE hydrogel. There are many factors that affect the tensile properties of polymers, such as molecular weight, crosslink density between polymer chains, crystallinity, testing velocity, temperature, and filler content. In this work, as previously reported, the molar masses of the macromers followed the order of PAHCM-AGE > PASCM-AGE > PACM-AGE. Macromer chains with higher molar masses are more entangled and less free to move, contributing to the higher tensile strength observed in PAHCM-AGE hydrogels compared to PASCM-AGE and PACM-AGE hydrogels. Additionally, the presence of the amide group in HEA can enhance intermolecular forces (through both hydrogen bonding and Van der Waals forces) between polymer chains, further promoting higher tensile strength; this is similar to other hydrogels that contain HEA [34]. Thus, there are two factors affecting this mechanical performance: the molar mass of the polymer and the molecular structure of the polymers. Additionally, as the macromers have the ability to form networks without the addition of an external crosslinker, PACM-AGE was selected and tensile performance assessed without any crosslinker. The non-crosslinked PACM-AGE showed high tensile elongation (ca. 142%) but very low tensile strength (ca. 0.05 MPa) when compared with other crosslinked hydrogels. This low strength results in a material that lacks elasticity to an applied force.

#### 3.2.3. Dye Release Profiles

**Figure 8:**
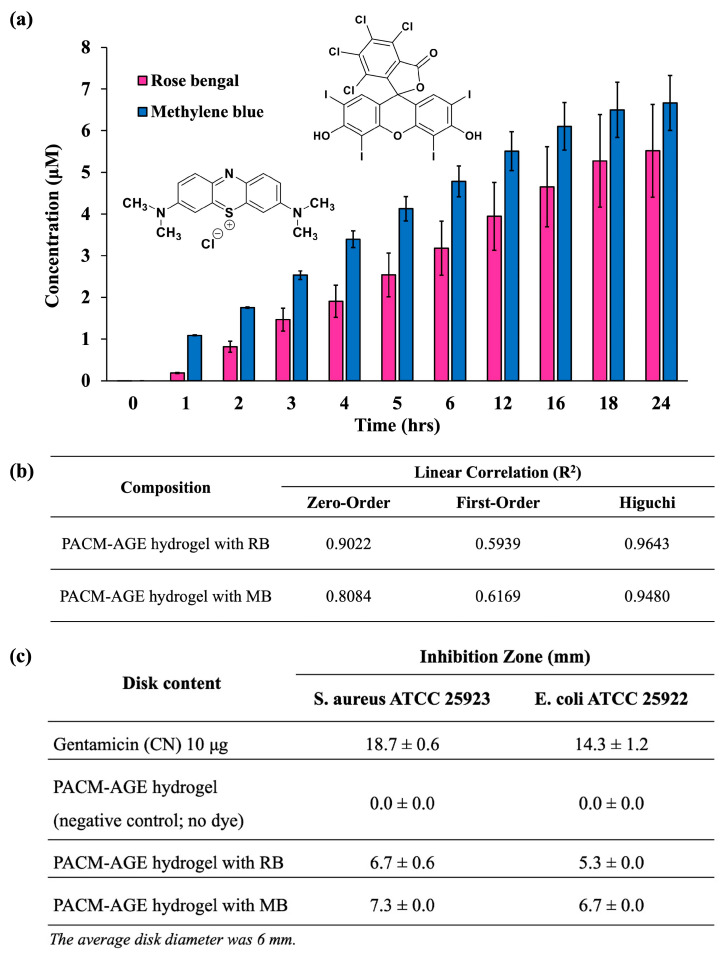
(**a**) Dye release profiles of PACM-AGE hydrogel sheet using Rose Bengal (RB) and Methylene Blue (MB) in PBS over 24 h, (**b**) table of linear correlation derived from kinetic models, and (**c**) table showing inhibition zone (mm) for both bacteria, Staphylococcus aureus (*S. aureus*) and Escherichia coli (*E. coli*), of hydrogels with RB and MB as photosensitizing agents.

To further explore the potential applications of hydrogels derived from macromers, we investigated the uptake and release of two water-soluble photosensitizing agents, Rose Bengal (RB) and Methylene Blue (MB), from PACM-AGE hydrogels. The cumulative dye release was calculated using standard calibration curves for both RB and MB. Results indicated that PACM-AGE hydrogels exhibited greater uptake of RB (79%) compared to MB (45%). The release profiles of both RB and MB from the PACM-AGE hydrogels showed a continuous increase over time, with MB consistently releasing at higher concentrations than RB at each observed time point (Figure 8a). Specifically, PACM-AGE hydrogels released RB in the range of 0.18 μM to 5.52 μM and MB in the range of 1.08 μM to 6.66 μM. This variation in release is likely attributed to the differences in molecular weight between RB (1017.64 g/mol) and MB (319.85 g/mol), as well as the specific interactions between the dyes and the macromer structure, influenced by their charges (RB being anionic and MB being cationic). To better understand the release kinetics, three release kinetic models were used to assess cumulative release: zero-order (cumulative release vs. time), first-order (log cumulative release vs. time), and the Higuchi model (cumulative release vs. square root of time). As shown in Figure 8b, the release of both RB and MB from PACM-AGE hydrogels best fit the Higuchi model, with linear correlation coefficients (R^2^) of 0.9643 for RB and 0.9480 for MB. This suggests that the release mechanism is primarily diffusion-controlled, consistent with the nature of these hydrogels.

#### 3.2.4. Anti-Bacterial Properties

The antibacterial activity of the dye, for both RB or MB, was examined by the disk diffusion method according to CLSI guidelines [35] and the diameter of the inhibition zone was observed. The microorganisms used in this study were *S. aureus* ATCC 25923, a representative of Gram-positive bacteria, whereas *E. coli* ATCC 25922 was representative of Gram-negative bacteria. Gentamicin and the PACM-AGE hydrogel without a photosensitizing agent were used as the positive and negative controls, respectively. To test the samples, hydrogels loaded with RB were exposed to green light at 532 nm, while hydrogels with MB were exposed to red light at 650 nm. The light exposure for both photosensitizing agents lasted for 5 min before the start of the antibacterial test. The antibacterial activity of RB and MB is primarily attributed to their function as photosensitizers. Upon exposure to specific wavelengths of light, RB and MB generate reactive oxygen species (ROS), such as singlet oxygen and free radicals. These ROS cause oxidative damage to bacterial cell membranes, proteins, and DNA, leading to cell death. This photodynamic effect is well-documented for both Gram-positive and Gram-negative bacteria, making RB and MB effective antibacterial agents when activated by light [36]. Figure 8c presents the inhibition zone data of the hydrogels (see ESI Figure S1 for agar plate photographs of inhibition zones). For *S. aureus*, PACM-AGE hydrogels with RB disks gave a mean inhibition zone of 6.7 ± 0.3 mm, while PACM-AGE hydrogels with MB gave a mean of 7.3 ± 0.9 mm. For *E. coli*, PACM-AGE hydrogels with RB disks gave a mean inhibition zone of 5.3 ± 0.4 mm, while PACM-AGE hydrogels with MB gave a mean of 6.7 ± 0.5 mm. The positive control gave inhibition zones of 18.6 ± 0.6 mm for *S. aureus* and 14.3 ± 1.2 mm for *E. coli*, while the negative control (PACM_AGE hydrogel, no dye) showed no inhibition zones for both bacteria. These results align with findings in the literature [37], indicating that RB and MB exhibit a dose-dependent effect on bacterial inhibition for both Gram-positive and Gram-negative bacteria. The negative control (PACM-AGE hydrogel without a photosensitizer) highlights that the hydrogel itself has no antibacterial activity. However, with the incorporation of RB and MB, these macromers can be used in applications that benefit from this behavior, such as wound dressing.

## 4. Conclusions

The novel AMPS-based macromers, PAHCM-AGE and PASCM-AGE, have been successfully synthesized to develop AMPS-based hydrogels. The co-macromers showed effective hydrogel formation with improved control over mechanical strength, swelling ratio, and water content. All results revealed that incorporating HEA or SPA enhanced mechanical performance and swelling behavior, preventing excessive water absorption and improving mechanical strength. PASCM-AGE hydrogels demonstrated superior tensile strength and elongation compared to the original macromer hydrogel. Additionally, PACM-AGE hydrogels with photosensitizers Rose Bengal (RB) and Methylene Blue (MB) exhibited light-activated antibacterial activity. While these are promising results, the limitation of requiring further in vivo studies is acknowledged to comprehensively evaluate the potential of the hydrogels as effective wound dressings in the future. In summary, PAHCM-AGE and PASCM-AGE hydrogels with enhanced properties were successfully developed, highlighting the importance of controlling water intake and mechanical properties for robust biomedical hydrogels.

## Figures and Tables

**Table 1:** Compositions of AMPS-based copolymers and macromers.

Code	Synthesized Samples	Macromers (mol eq.)				Reactants (mol eq.)			
		AMPS (A)	HEA (H)	SPA (S)	CEA (C)	MAA (M)	APS	AGE	Zn
	Copolymers								
PACM	Poly(AMPS-*stat*-CEA-*stat*-MAA)	1	-	-	0.4	0.4	0.02	-	-
PAHCM	Poly(AMPS-*stat*-HEA-*stat*-CEA-*stat*-MAA)	0.75	0.25	-	0.4	0.4	0.02	-	-
PASCM	Poly(AMPS-*stat*-SPA-*stat*-CEA-*stat*-MAA)	0.75	-	0.25	0.4	0.4	0.02	-	-
	Macromers								
PACM-AGE	Poly(AMPS-*stat*-CEA-*stat*-MAA)-*graft*-AGE	1	-	-	0.4	0.4	0.02	0.8	0.06
PAHCM-AGE	Poly(AMPS-*stat*-HEA-*stat*-CEA-*stat*-MAA)-*graft*-AGE	0.75	0.25	-	0.4	0.4	0.02	0.8	0.06
PASCM-AGE	Poly(AMPS-*stat*-SPA-*stat*-CEA-*stat*-MAA)-*graft*-AGE	0.75	-	0.25	0.4	0.4	0.02	0.8	0.06

## Data Availability

The data presented in this study are available on request from the corresponding author. The data are not publicly available due to the data forms part of an ongoing study.

## Supplementary Materials

The following supporting information can be downloaded at https://www.mdpi.com/article/10.3390/polym16172522/s1. Figure S1: Agar plate examples from antibacterial testing with *Staphylococcus aureus* (*S. aureus*) (a–d) and *Escherichia coli* (*E. coli*) (e–h). (a,e) Positive control (10 mg gentamicin); (b,f) negative control (PACM-AGE hydrogel without dye); (c,g) PACM-AGE hydrogel with Rose Bengal (RB); (d,h) PACM-AGE hydrogel with Methylene Blue (MB).
