# Supplementary material for: Tailoring Hydrogel Sheet Properties through Co-Monomer Selection in AMPS Copolymer Macromers
Source: Polymers (Basel). 2024 Sep 5;16(17):2522. doi: 10.3390/polym16172522 (PMC11397928; doi:10.3390/polym16172522)
Supplement: Supplementary file 1 [file polymers-16-02522-s001.zip › polymers-3156247-supplementary.pdf]

Tailoring Hydrogel Sheet Properties through Co-Monomer Selection in AMPS Copolymer Macromers

Jinjutha Daengmankhong[1], Thanyaporn Pinthong[1], Sudarat Promkrainit[1], Maytinee Yooyod[1], Sararat Mahasaranon[1,2], Winita Punyodom[3,4], Sukunya Ross [1,2], Jirapas Jongjitwimol[2,5], Brian J. Tighe[6], Matthew J. Derry[6], Paul D. Topham[6], Gareth M. Ross[1,2,*]

Electronic Supporting Information (ESI)

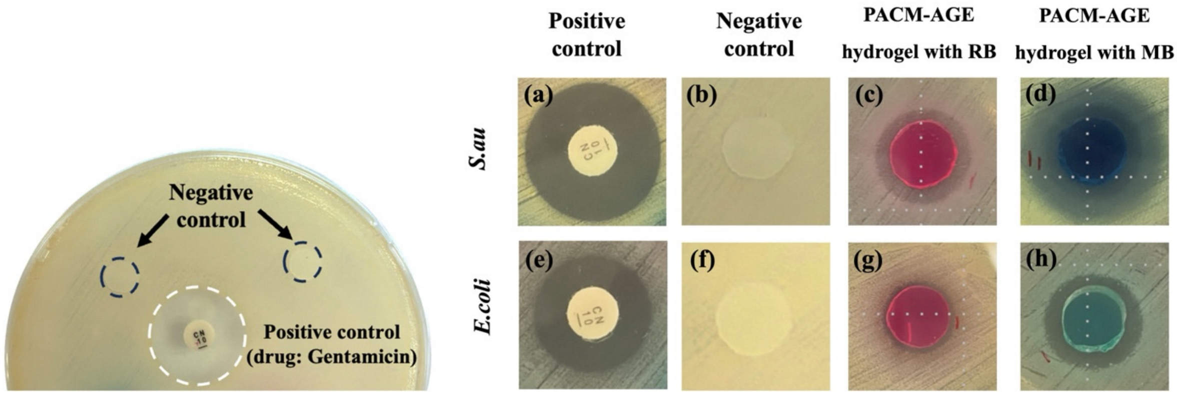

Figure S1. Agar plate examples from antibacterial testing with *Staphylococcus aureus* (S. aureus) (a-d) and *Escherichia coli* (E. coli) (e-h). (a, e) Positive control (10 mg gentamicin); (b, f) negative control (PACM-AGE hydrogel without dye); (c, g) PACM-AGE hydrogel with Rose Bengal (RB); (d, h) PACM-AGE hydrogel with Methylene Blue (MB).

Fig. S1 shows the antibacterial tests of hydrogel samples against *Staphylococcus aureus* (*S. aureus*) ATCC 25923 (Fig. S1a-d) and *Escherichia coli* (*E. coli*) ATCC 25922 (Fig. S1e-h). The positive control (10 mg gentamicin) produced inhibition zones of 18.6 ± 0.6 mm for *S. aureus* and 14.3 ± 1.2 mm for *E. coli*, while the negative control (PACM-AGE hydrogel without dye) showed no inhibition zones. For *S. aureus*, PACM-AGE hydrogels with rose bengal (RB) had a mean inhibition zone of 6.7 ± 0.3 mm, and those with methylene blue (MB) had 7.3 ± 0.9 mm. For *E. coli*, PACM-AGE hydrogels with RB had a mean inhibition zone of 5.3 ± 0.4 mm, and those with MB had 6.7 ± 0.5 mm.
